# Close Encounters of the Cell Kind: The Impact of Contact Inhibition on Tumour Growth and Cancer Models

**DOI:** 10.1007/s11538-019-00677-y

**Published:** 2020-01-22

**Authors:** David Robert Grimes, Alexander G. Fletcher

**Affiliations:** 1 School of Physical Sciences, Dublin City University, Glasnevin, Dublin 9, Ireland; 2 Department of Oncology, University of Oxford, Old Road Campus, Oxford, OX3 7DQ UK; 3 School of Mathematics and Statistics, University of Sheffield, Sheffield, S3 7RH UK; 4 Bateson Centre, University of Sheffield, Sheffield, S10 2TN UK

**Keywords:** Tumour growth, Cancer, Growth laws, Mathematical oncology

## Abstract

Cancer is a complex phenomenon, and the sheer variation in behaviour across different types renders it difficult to ascertain underlying biological mechanisms. Experimental approaches frequently yield conflicting results for myriad reasons, and mathematical modelling of cancer is a vital tool to explore what we cannot readily measure, and ultimately improve treatment and prognosis. Like experiments, models are underpinned by certain biological assumptions, variation of which can lead to divergent predictions. An outstanding and important question concerns contact inhibition of proliferation (CIP), the observation that proliferation ceases when cells are spatially confined by their neighbours. CIP is a characteristic of many healthy adult tissues, but it remains unclear to which extent it holds in solid tumours, which exhibit regions of hyper-proliferation, and apparent breakdown of CIP. What precisely occurs in tumour tissue remains an open question, which mathematical modelling can help shed light on. In this perspective piece, we explore the implications of different hypotheses and available experimental evidence to elucidate the implications of these scenarios. We also outline how erroneous conclusions about the nature of tumour growth may be arrived at by looking selectively at biological data in isolation, and how this might be circumvented.

## Introduction

Cancer is a deeply complex phenomenon, and mathematical modelling has become a powerful and increasingly important tool in cancer research (Byrne 2010; Anderson and Quaranta 2008). It provides an *in silico* laboratory to investigate hypothesized mechanisms of cancer progression and predict the response to different interventions. Mathematical models can readily inform *in vivo* and *in vitro* experiments and predict previously unseen behaviour. Equally, they can be informed by biological data to yield more robust conclusions. Used correctly, modelling can both identify interesting avenues for future research and streamline the design of new experiments, thus contributing to the 3Rs principles of animal experimentation (replacement, refinement and reduction). Mathematical modelling of solid tumour growth has long been an area of interest, and a multitude of mathematical models derived to capture different aspects of tumour growth (Gerlee 2013), including heterogeneity, treatment response and interactions with host tissues.

Yet models, like experiments, are underpinned by assumptions. There are emerging biological data which suggest that cells in two-dimensional configurations behave markedly different than those in three-dimensional aggregates (Pickl and Ries 2009; Kunz-Schughart et al. 2000; Edmondson et al. 2014; Imamura et al. 2015; Riedl et al. 2016; Stock et al. 2016). Accordingly, model assumptions that are suitable for healthy tissue or a particular cancer type may not be applicable in other circumstances. It is important also to distinguish between a phenomenological description, whose parameters may have no direct physical correlate, and a mechanistic model that seeks to describe the underlying physical processes (Tracqui 2009; Araujo and McElwain 2004).

Conflicting experimental findings are common too, and accordingly interpretation and extrapolation of experimental results is also fraught with difficulty. Solid tumour growth dynamics illustrate this point well. Historically, tumour growth has been described by sigmoidal functions, including the von Bertalanffy, Gompertzian and logistic family of models (Steel 1977; Wheldon 1988; Vaidya and Alexandro 1982). In these models, growth is initially unrestrained, before becoming limited by depletion of essential nutrients such as oxygen, with approximately sigmoidal functions generally thought of as adequate to describe general avascular growth (Feller 1940; Gyllenberg and Webb 1988; Marušić et al. 1994). On the other hand, it has been suggested based on colony evidence that tumour growth is not limited by nutrient availability, but by spatial constraints (Brú et al. 2003), such that tumour radius grows linearly with time, and is restricted to the periphery. This claim remains controversial (Buceta and Galeano 2005), but serves as a prominent example of conflicting claims in the literature. In addition, there is often unavoidable ambiguity in available biological data, which may be of unclear provenance. This can result in situations where biological data may be incorrectly interpreted as providing evidence in support of a modelling prediction when this may not be the case.

Biologically, these divergent views can be recast as a question of whether cancers in general remain subject to contact inhibition of proliferation (CIP). In healthy tissues, cell proliferation is inhibited as a result of cell-cell contact (Nelson and Chen 2002; Holley and Kiernan 1968; Harry and Levine 1967). While precise mechanisms are not yet fully understood, the signalling pathways underlying CIP in adult tissues are starting to be elucidated (Küppers et al. 2010), with evidence for the involvement of the rapamycin (Leontieva et al. 2014) and hippo pathways (Zeng and Hong 2008). This suggests that only cells on the tissue periphery can undergo mitosis. However, hyper-proliferation is a hallmark of cancer (Hanahan and Weinberg 2000) and it is important to probe potential reasons for this. There is experimental evidence for failure of normal CIP mechanisms in human cancers (Levine et al. 1965; Kim et al. 2004; Lloyd et al. 1999; Leontieva and Blagosklonny 2011; McClatchey and Yap 2012), while studies on the naked mole rat have suggested that the animal’s remarkable apparent immunity to cancer may be related to its hyper-sensitivity to cell–cell inhibition (Seluanov et al. 2009). This suggests that CIP is greatly reduced or absent in many solid tumours. In these cases, cells that would normally be unable to proliferate in healthy tissue due to their spatial location become able to undergo mitosis.

This is an important consideration, as spatial localization of proliferation affects our predictions on tumour growth and response to treatment. Precisely what is occurring remains unclear, but here we argue that mathematical models can shed some light on predicted behaviour, demonstrating that CIP is a good example of an instance where modelling can help resolve debates in biology. Here, we probe the predictions and implications of both paradigms in 2D plated cells and 3D avascular tumours. The impact of these different assumptions are simulated, and compared with experimental data. The biological and modelling implications stemming from this analysis are discussed, and future avenues to better elucidate the problem explored.

## Methods

### Analysis of 2D Plated Cell Growth

Plated cell monolayers remain the simplest way to examine cell growth *in vitro*, with no nutrient heterogeneity so that all cells receive ample glucose and oxygen. Under the assumption of CIP, only cells at the edge of a cell colony proliferate. Assuming an average cell diameter of *L*, average doubling time $t_\mathrm{d}$ and initial colony radius of $r_{0}$, the area $a_\mathrm{c}$ of a circular colony under CIP, and therefore grows quadratically with time *t*:

1 $$\begin{aligned} a_\mathrm{c}(t) = \pi \left( r_{0} + \frac{Lt}{t_\mathrm{d}} \right) ^2. \end{aligned}$$

Without the constraint of CIP, the area instead grows exponentially:

2 $$\begin{aligned} a_{u}(t) = (\pi r_{o}^2) \, 2^{t/t_d}. \end{aligned}$$

The growth dynamics predicted for 2D plated cell colonies are not especially useful for gaining insight into three-dimensional tumour growth, given their implicit assumption of nutrient homogeneity. Even so, it is important to quantify potential differences that would be expected in growth dynamics with or without CIP.

### Analysis of 3D Avascular Tumour Growth

Multicellular tumour spheroids are the simplest of 3D cellular aggregates, extensively employed to study tumour growth dynamics, as their growth dynamics more closely resemble those of in situ tumours than do monolayer cultures. Such assays have been widely used in experimental and modelling studies (Hirschhaeuser et al. 2010). As spheroids grow, central regions become devoid of essential nutrients such as oxygen, and as a consequence tumour spheroids develop regions of central hypoxia and eventually necrosis, just as in avascular tumours. The extent of central necrosis and the oxygen distribution throughout the spheroid depends upon the oxygen consumption rate of the cell line in question (Grimes et al. 2014a, b).

Growth dynamics for multicellular tumour spheroids have been well-studied (Conger and Ziskin 1983; Freyer 1988; Grimes et al. 2016). Evidence suggests that spheroids exhibit a classical sigmoidal growth profile. Conger and Zisikin (1983) examined spheroid growth over multiple cell lines, finding that spheroids have an initial exponential growth phase, followed by a quasi-linear phase where limited nutrient diffusion inhibits growth, and finally a plateau phase. Such dynamics are similar to growth curves exhibited by solid tumours in situ (Steel 1977; Conger and Ziskin 1983; Gyllenberg and Webb 1988; Grimes et al. 2016). The Gompertzian model captures tumour growth dynamics especially well, but can lead to unrealistically slow growth in initial phrases. Wheldon Wheldon (1988) proposed a hybrid ‘Gomp-ex’ model to better capture early growth behaviour, also reflected in tumour growth dynamics (Benzekry et al. 2014).

Multicellular tumour spheroids present an excellent test bed for examining CIP assumptions. Broadly speaking, there are two possible scenarios: if we assume that CIP is in effect, then for a initial small spheroid only cells on the outermost layer proliferate, while those inside the central mass are inhibited from mitosis. Assuming spherical symmetry, if cells have an average diameter *L* and average doubling time $t_\mathrm{d}$, then the change in radius over time is given by $\mathrm{d}r/\mathrm{d}t = L/t_\mathrm{d}$. Defining the initial radius to be $r_{0}$, we find that the spheroid radius is given by $r(t) = r_{0} + Lt/t_\mathrm{d}$, and thus its volume $V_\mathrm{c}(t)$ is given by

3 $$\begin{aligned} V_\mathrm{c}(t) = \frac{4\pi }{3}\left( r_{0} + \frac{Lt}{t_\mathrm{d}} \right) ^3. \end{aligned}$$

Thus, under CIP assumptions, a cubic growth rate essentially agnostic to the internal nutrient distribution is expected, with cells on the external border continuing to grow (Brú et al. 2003).

In contrast, if we assume CIP is defective in tumour cells, then any cell with enough nutrients will attempt to undergo mitosis. For spheroids grown *in vitro*, glucose levels are high throughout and oxygen availability is usually the limiting factor (Hirschhaeuser et al. 2010; Grimes et al. 2014b). There are various avascular growth models which can be employed to describe this (Roose et al. 2007); for simplicity, we take a simple recursive model that explicitly relates spheroid growth to nutrient availability (Grimes et al. 2014a). In this schema, the spheroid volume $V_{u}$ at time step $N+1$ is given by

4 $$\begin{aligned} (V_{u})_{N+1} = \frac{4\pi }{3} \left( 2 r_{N}^3 - (r_\mathrm{p})_{N}^3 - (r_{n})_{N}^3 \right) , \end{aligned}$$

where $r_{N}$ and $(r_{n})_{N}$ are the spheroid radius and necrotic radius at time step *N*, respectively, and $(r_\mathrm{p})_{N}$ is the radius below which the oxygen partial pressure *p* drops below the level required for mitosis, $p_{m}$. In practice, cells can undergo mitosis at even very low oxygen partial pressures, and typically $p_{m} \approx 0.5$ mmHg (Hockel and Vaupel 2001). All these radii can be analytically calculated from first principles knowing cellular oxygen consumption rate, with details omitted here for brevity (Grimes et al. 2016). In the case of unlimited nutrient diffusion, $r_{n} = r_\mathrm{p} = 0$, and growth is exponential.

### Model Comparisons with Experimental Data

*2D monolayers* For 2D growth, we simulate a simple cellular automaton model of tumour growth using Chaste (Osborne et al. 2017), an open-source C++ library for agent-based simulation of cell populations. Further details of the simulations are provided below.

In this model, cell movement is driven by division and cell exchange, using a shoving-based approach (Yates et al. 2015). The spatial domain is discretized into a regular square lattice with cells occupying the individual lattice sites. The area $A_{i}$ of each cell *i* in this model is given by 1 squared cell diameter (CD$^{2}$). Cell proliferation proceeds as follows: A dividing cell selects a random lattice site from its Moore neighbourhood (the eight cells that surround it), and all cells along the row, column or diagonal from the dividing cell’s location are instantaneously displaced or ‘shoved’ to make space for the new cell.

A Metropolis–Hastings algorithm is used to make additional updates to the state of the tissue using asynchronous updating. At each time step $\varDelta t$, after checking for and implementing any cell divisions, we sample with replacement $N_\mathrm{C}$ cells, where $N_\mathrm{C}$ is the number of cells in the tissue at time *t* (thus, it may be the case that a cell is sampled more than once in a time step, while others are not sampled). This sweeping of the domain is also known as a Monte Carlo step (MCS). We randomly select a neighbouring lattice site from each sampled cell’s Moore neighbourhood for a potential swap. The swapping of cells is intended to model random motility and the affinity of cells to form and break connections with adjacent cells. Assigning the MCS to a time step $\varDelta t$ allows us to parameterize the timescale of the switching process and relate this to cell division. A probability per hour is assigned for the cells (or empty lattice site, which we refer to as a void) to swap locations, $p_\mathrm{swap}$, which is calculated as

5 $$\begin{aligned} p_\mathrm{swap} = {\left\{ \begin{array}{ll} \kappa _\mathrm{swap},\quad \text{ for } \quad \varDelta H \le 0,\\ \kappa _\mathrm{swap}\exp \left( -\frac{\varDelta H}{T}\right) ,\quad \text{ for } \quad \varDelta H > 0. \end{array}\right. } \end{aligned}$$

where $\kappa _\mathrm{swap}$ represents the rate of switching and *T* represents the background level of cell switching, modelling random cell fluctuations. If $T=0$ then only energetically favourable swaps happen, and we use this as the default value for our simulations; as *T* increases, more energetically unfavourable swaps occur. Finally, $\varDelta H = H_{1} - H_{0}$ denotes the change in adhesive energy due to the swap, with $H_{0}$ and $H_{1}$ being the energy in the original and changed configurations, respectively, which is defined to be the sum of the adhesion energy between lattice sites:

6 $$\begin{aligned} H = \sum _{(i,j)\in {\mathcal {N}}}\gamma (\tau (i),\tau (j)), \end{aligned}$$

where $\gamma (a,b)$ is a constant whose value depends on *a* and *b*, representing the adhesion energy between cells (or void) of type *a* and *b*, $\tau (k)$ is the type of cell *k* (or void if there is no cell on the lattice site) and ${\mathcal {N}}$ is the set of all neighbouring lattice sites. Here, $\tau (k)$ takes the values ‘A’, ‘B’ and ‘void’, but can in principle be extended to more cell types.

In the 2D monolayer case, cell growth was simulated with and without CIP. To capture CIP failure, the cell-pushing was enabled, whereby cells to push their neighbours during mitosis. Resultant growth curves were obtained, and compared to the analytic growth curves given by Eqs. (1)–(2) and to the literature. Outputs of these models were then compared with data from the experimental literature to ascertain which model most faithfully reproduced observed dynamics.

*3D avascular tumour spheroids* We can readily investigate predictions for 3D tumour growth dynamics with and without CIP by analysing spheroid growth curves. Growth curves were generated analytically through the forms outlined in Eqs. (3)–(4), and these simulated spheroid growth curves was compared to previously published long-range data (over 60 days) (Freyer 1988; Marušić et al. 1994; Grimes et al. 2016) to ascertain model best fits under both assumptions. These growth dynamics are useful but they do not strictly answer the question of where proliferating cells are located in situ. To probe this directly, we interrogated histological specimens stained with Ki-67, a proliferative marker (Scholzen and Gerdes 2000). We looked at stained sections from tumour spheroid cross sections, which are broadly radially symmetric and relatively easy to interpret.

**Fig. 1:**
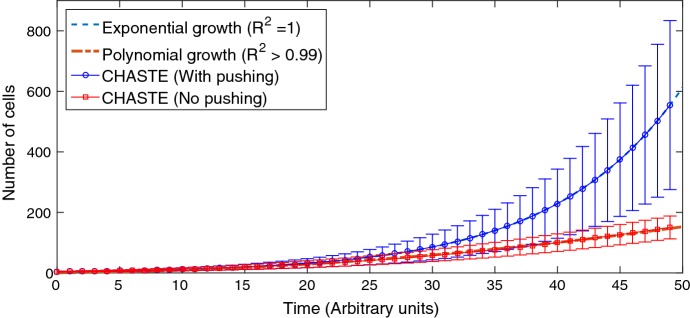
Chaste data (average and standard deviations obtained from 500 runs) with pushing (CIP failure) and without (CIP). In former case, the exponential form in Eq. (4) fits perfectly with $R^2 = 1$. In the latter, the polynomial expression in Eq. (3) fits with $R^2 >0.99$ (Color figure online)

## Results

### 2D Monolayers

As shown in Fig. 1, Chaste simulations without pushing (corresponding to a CIP assumption) produced quadratic polynomial fits in strong agreement with analytical form in Eq. (3). By contrast, allowing pushing produced solutions in agreement with the no CIP analytical model, which yields exponential growth as predicted by Eq. (4). This latter scenario agrees with the bulk of published literature of 2D monolayers, where exponential growth is typically observed (Demicheli et al. 1989; Sutherland et al. 1983; Erlichman and Vidgen 1984; Wheldon 1988; Steel 1977).

**Fig. 2:**
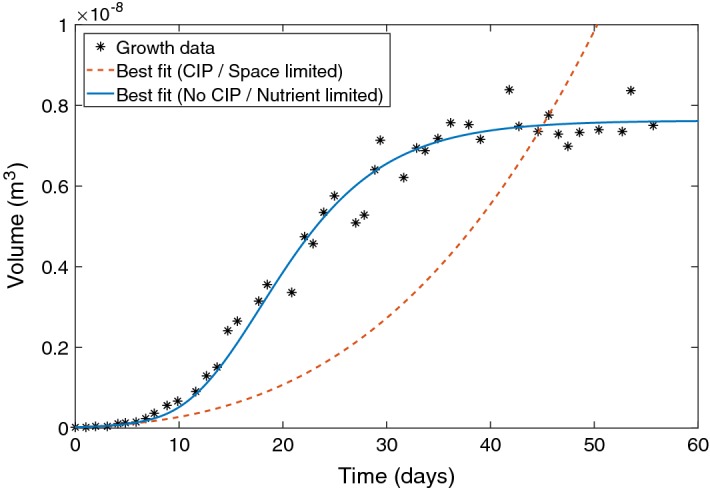
Best-fit growth curves for analytical models relative to tumour spheroid data (Freyer 1988) assuming either CIP (space-limited) or no CIP (nutrient limited) scenarios. The assumption of CIP yielded a best-fit with a negative coefficient of determination ($L/t_\mathrm{d} = 2.314 \times 10^{-5}$ m/day), indicating this does not describe the data at all. By contrast, the mechanistic model assuming no CIP yielded excellent fit ($R^{2} = 0.9939$) with parameters that were biologically realistic (Color figure online)

### 3D Avascular Tumour Spheroids

**Fig. 3:**
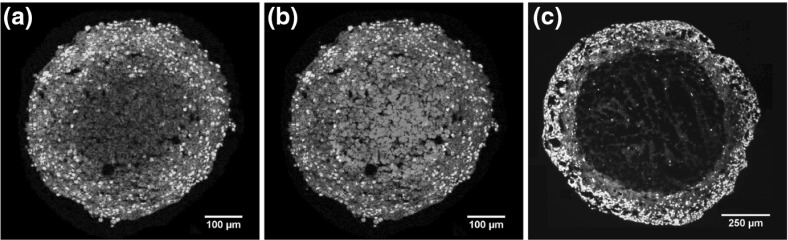
**a** HCT-116 tumour spheroid stained with Ki-67 (green), a marker of proliferation grown for 4 days. **b** The same spheroid co-stained with the hypoxia marker EF5 (red). Proliferation is apparent throughout the entirety of the spheroid, while there is no central region of anoxia. Images reproduced with permission (Grimes et al. 2016). **c** Dual-stained DLD-1 tumour spheroid with central necrosis showing Ki-67 (green) and EF5 (red) grown for 12 days. Proliferation occurs throughout the viable rim. Reproduced with permission (Grimes et al. 2014b) (Color figure online)

For the available long-range spheroid data, best-fit parameters were found for both CIP and no CIP assumptions. Best-fit parameter values are given in Fig. 2, illustrated with results. Assuming CIP in this instance yields a negative coefficient of determination, which means the fit was worse than merely fitting the mean. This strongly suggests that such a model is inadequate to describe the growth data. By contrast, the no CIP assumption fitted the data well ($R^2 = 0.9939$) and yielded biologically realistic values for oxygen consumption rate ($a = 6.87$ mmHg) and cellular doubling time $t_\mathrm{d} = 2.18$ days. As similar patterns of growth are seen throughout spheroid derived from many different cell lines (Conger and Ziskin 1983; Wheldon 1988; Marušić et al. 1994; Grimes et al. 2016), this suggests that CIP in inhibited in these cell lines, and that growth is not restricted to the outermost extremities. Previously published datasets from sectioned and stained tumour DLD-1 (Grimes et al. 2014b) and HCT-116 (Grimes et al. 2016) tumour spheroids were analysed to determine the extent of Ki-67 staining. Figure 3 depicts sectioned some of these tumour spheroids stained with Ki-67 proliferation marker—in all cases, evidence of mitosis is seen throughout the spheroids, and not solely at the boundaries. This strongly suggests that CIP is absent from these spheroids. In principle, the model outlined in Eq. (4) could serve to model both CIP and no CIP assumptions, where the proliferation radius $r_\mathrm{p}$ would be markedly reduced. This would then produce curves with similarly implausible biological parameter values. Equation (3) has been presented to explicitly depict the CIP scenario. Strictly speaking, this is always a simplification, as there will always been some diffusion limit where the growth will be ultimately saturated (Bodnar and Foryś 2007), and thus infinite growth would never be observed.

**Fig. 4:**
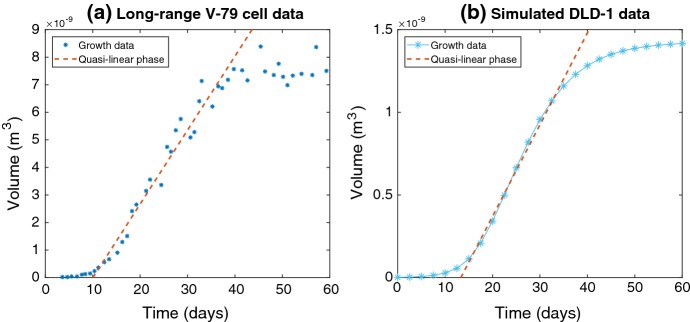
**a** Long-range growth data for V-79 hamster cells taken from Freyer et al. (1988), depicted with a linear fit through the quasi-linear growth phase with $R^2 > 0.96$. **b** Simulated growth of a DLD-1 tumour spheroid using a mechanistic growth model (Grimes et al. 2016), with a linear fit of $R^2 > 0.99$ through the quasi-linear phase (Color figure online)

## Discussion

The assumption that only cells on the periphery undergo mitosis seems to be contradicted by experimentally derived growth curves, with histological data suggesting that mitotic activity occurs in the tumour mass itself too. Even so, we must be careful not to overstate the generality of these conclusions, as it is entirely possible that different cell lines have varying extents of CIP. In some immortalized cell lines, for example, CIP may still occur despite these cells having the ability to proliferate indefinitely (Abercrombie 1979). As the precise mechanisms for CIP are not fully understood, further experimental evidence will be vital in illuminating this area.

In addition, there are some important caveats to this conclusion, and avenues for further research. While the evidence presented here suggests cellular proliferation is not solely limited to the edge of a tumour or colony, one thing not considered thus far is the mechanical constraints on a neoplasm. In general, tumours are physically constrained to a position inside the body. A tumour growing in situ might not have contact inhibition, but instead might be eventually be physically limited due to hard boundaries in the form of tissue or organs. This would manifest especially in tissue resilient to deformation, including bone (Araujo et al. 2014).

More importantly perhaps, even with CIP-inhibition cells cannot ‘infinitely push’—while all cells with sufficient nutrients and clonogenic capacity might be able to undergo mitosis, there is likely a point where the surrounding density of cells is so high that mechanical pressures alone arrest the cell’s mitotic phase and force it into quiescence. In healthy animal cells, forces $> 100$nN were sufficient to impinge on microtubule spindle function and thereby inhibit mitotic progression (Cattin et al. 2015), with similar trends seen in mechanically compressed spheroids (Desmaison et al. 2013).When tumour growth is limited by mechanical forces acting on the cells and effectively constrained, then a phenomenological treatment of this as equivalent to CIP to capture the behaviour of the system seems an appropriate assumption. The interpretation of such models will then depend on whether the parameters within are considered biologically realistic or are intended to explore specific phenomena.

In real settings of course, tumours do not simply grow into empty space, but within a tissue. As a result, a form of CIP likely takes place at the tumour periphery, the effects of which are not considered in the simple models discussed in this work. There are other potentially obscuring factors in real tumours; for example, some tumours might exhibit high cellular turnover rates, and even if tumours did display relatively high levels of CIP, the density would still on average be lower, and proliferation less inhibited. Conversely, if cell death was high, then proliferation might still be seen in stained sections even if CIP was intact. These are certainly worthwhile questions beyond the scope of this work, and ones that require combined clinical/experimental and theoretical investigation to adequately probe.

It is worth noting the conflict between Brú’s paradigm (Brú et al. 2003) for linear ’universal’ tumour growth versus the sigmoidal growth more typically reported by investigators. There are many reasons why these results may be in conflict—firstly, it is a reality that experimental data are notoriously difficult to replicate. Cancer research is complicated by the sheer number of confounding factors possible, and similar experiments done in different laboratories can yield entirely different results for reasons not entirely understood. It is worth noting that most of the experimental results in this work were derived from 2D culture, and thus may not extrapolate to 3D, although in general 2D culture growth curves are not linear. While growth curves in 3D are not generally linear, there might be a further way to explain the discrepancy. There is a substantial region where growth is effectively linear, known as the quasi-linear growth phase (Conger and Ziskin 1983). Measurements taken in this regime will suggest an effectively linear rate of growth, and occur in all sigmoidal models. This is shown in Fig. 4 for both biological data and simulated mechanistic growth.

The data and analysis presented here suggests that CIP is in general a casualty of oncogenesis, and potentially a target for future therapy. The extent to which this is generalizable remains unanswered, and to truly discover, the underlying physical mechanisms shaping growth dynamics will demand a much more comprehensive synthesis of experimental data with modelling approaches. We believe that combined clinical/experimental and theoretical approaches (Anderson and Quaranta 2008) hold the greatest chance of unravelling this mystery. Answering this question will improve our understanding of how cancer perpetuates, and potentially yield new insights into how we combat it.
